# Comparison of contrast enhanced three dimensional echocardiography with MIBI gated SPECT for the evaluation of left ventricular function

**DOI:** 10.1186/1476-7120-7-27

**Published:** 2009-06-16

**Authors:** Bernard Cosyns, David Haberman, Steven Droogmans, Sandrine Warzée, Philippe Mahieu, Eric Laurent, Marie Moonen, Sophie Hernot, Patrizio Lancellotti

**Affiliations:** 1 CHIREC, Cardiology department, Free University of Brussels, Belgium; 2 UZ Brussel, Cardiology department, Vrije Universiteit van Brussel, Belgium; 3 CHU Tivoli, Cardiology department, Free University of Brussels, Belgium; 4 CHIREC, Nuclear Medicine, Free University of Brussels, Belgium; 5 CHU Sart Tilman, Cardiology department, University of Liège, Belgium

## Abstract

**Background:**

In clinical practice and in clinical trials, echocardiography and scintigraphy are used the most for the evaluation of global left ejection fraction (LVEF) and left ventricular (LV) volumes. Actually, poor quality imaging and geometrical assumptions are the main limitations of LVEF measured by echocardiography. Contrast agents and 3D echocardiography are new methods that may alleviate these potential limitations.

**Methods:**

Therefore we sought to examine the accuracy of contrast 3D echocardiography for the evaluation of LV volumes and LVEF relative to MIBI gated SPECT as an independent reference. In 43 patients addressed for chest pain, contrast 3D echocardiography (RT3DE) and MIBI gated SPECT were prospectively performed on the same day. The accuracy and the variability of LV volumes and LVEF measurements were evaluated.

**Results:**

Due to good endocardial delineation, LV volumes and LVEF measurements by contrast RT3DE were feasible in 99% of the patients. The mean LV end-diastolic volume (LVEDV) of the group by scintigraphy was 143 ± 65 mL and was underestimated by triplane contrast RT3DE (128 ± 60 mL; p < 0.001) and less by full-volume contrast RT3DE (132 ± 62 mL; p < 0.001). Limits of agreement with scintigraphy were similar for triplane andfull-volume, modalities with the best results for full-volume. Results were similar for calculation of LV end-systolic volume (LVESV). The mean LVEF was 44 ± 16% with scintigraphy and was not significantly different with both triplane contrast RT3DE (45 ± 15%) and full-volume contrast RT3DE (45 ± 15%). There was an excellent correlation between two different observers for LVEDV, LVESV and LVEF measurements and inter observer agreement was also good for both contrast RT3DE techniques.

**Conclusion:**

Contrast RT3DE allows an accurate assessment of LVEF compared to the LVEF measured by SPECT, and shows low variability between observers. Although RT3DE triplane provides accurate evaluation of left ventricular function, RT3DE full-volume is superior to triplane modality in patients with suspected coronary artery disease.

## Background

The determination of the global LV function and cardiac volumes remains one of the most important issues in the everyday practice of the cardiologist because these measurements are shown to correlate most strongly with prognosis among patients with cardiac disease [1,2]. Due to its high spatial and temporal resolution, 2D echocardiography (2DE) is widely used for the determination of global and regional ventricular function. However, its accuracy is limited significantly when underlying cardiac pathology is present [3-5]. The advantage of scintigraphy is that the acquisition of the images is much less observer-dependent than in echocardiography. Moreover, non-echocardiogaphic methods work in patients in whom ultrasound methods fall because of a poor acoustic window. Scintigraphy and angiography have been validated in numerous trials. Nuclear gated blood-pool imaging is considered the gold standard for the assessment of LV function because it provides a 3 D data set. Scintigraphy is reproducible and reliable for the assessment of parameters of global cardiac function (LVEF, LVESV and LVEDV) and widely available, but is associated with limitations that also preclude routine application [6,7] and is relatively costly for the data obtained. It is now used more as a reference method to validate new echocardiographic methods [8]. In clinical practice there is no real alternative to echocardiography, which is widely available, portable and repeatable.

RT3DE technology, which has recently become widely available, allows fast acquisition from a single acoustic window of dynamic data that encompass the entire heart. Using 3D imaging, less geometrical assumptions are made, and thus RT3DE should be more useful for the assessment of left ventricular size and function. Several recent studies have demonstrated the potential improvements in the evaluation of global LV function from RT3DE data [9]. One important limitation of echocardiography remains the inability to define the endocardial border in a subset of patients with poor image quality, therefore limiting the feasibility of LVEF measurements and its reproducibility in a non selected population [10]. Over the past decade, the use of contrast agents has been successfully applied to overcome this limitation [11] with 2DE and more recently with RT3DE. To date, this combination of contrast with RT3DE has been compared to unenhanced RT3DE or with magnetic resonance imaging (MRI) [12,13], but no data regarding comparison with MIBI gated SPECT are available. MRI can not be used in daily clinical practice and in large clinical trials. Only echocardiography and nuclear cardiology permit the determination of both parameters on a regular basis.

Therefore, we aimed to compare the feasibility and the accuracy of contrast enhanced RT3DE triplane and RT3DE full-volume with MIBI gated SPECT, as an independent reference, for the evaluation of the LVEF and cardiac volumes.

## Methods

### Study design

We prospectively recruited 43 consecutive patients addressed for chest pain to the echocardiography laboratory for measurement of LV volumes and LVEF. After exclusion of one patient with poor echocardiographic images and three with atrial fibrillation, a study group of 38 patients (20 men, 18 women; age, 70 ± 11 years) who underwent contrast RT3DE and MIBI gated SPECT during the same day within one hour.

### Real-time three-dimensional echocardiography

#### A. Triplane

**Figure 1:**
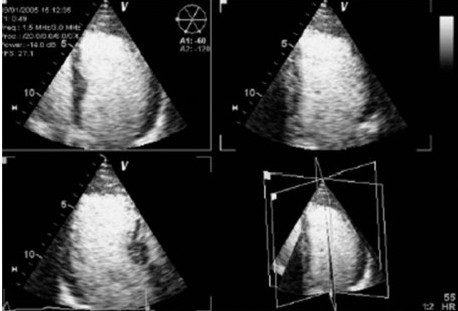
**Example of contrast RT3DE triplane showing the apical three orthogonal cutting planes**.

Enhanced contrast studies were performed with a commercially available echocardiographic platform (VIVID 7, GE Vingmed Ultrasound, Horten, Norway), equipped with a 3V-probe for triplane acquisition in harmonic mode. Patients were scanned in the left lateral decubitus position, from the apical window. Care was taken to visualise the true LV apex allowing simultaneous acquisition of the apical four-, two- and three-chamber views. (Figure 1) Cineloops of 3 cardiac cycles of the triplane quadscreen, were digitally stored in raw data format. Sector size and depth were adjusted to achieve optimal visualisation of the six LV walls at the highest possible frame rate. During post-processing, the triplane dataset was frozen in end diastole and the endocardial border was manually traced in the apical four-, two- and three-chamber, views respectively. To enhance endocardial border delineation, the software allows separate visualisation of the three apical views. Then, using the same heartbeat, the triplane dataset was frozen in end systole and again the endocardial border was manually traced in the apical four-, two- and three-chamber views. Papillary muscle was systematically left within the cavity. A 3-D LVEDV and LVESV was generated automatically by the software and LV volumes and ejection fraction were reported accordingly [see Additional file 1]. The measurements from the 3 consecutive beats recorded were averaged.

#### B. Full-volume

RT3DE full-volume images with contrast were also obtained from an apical window with the patient in the same position. Images were gathered over 4 cardiac cycles using the same matrix-array ultrasonographic transducer. Measurements of RT3DE volumes were performed offline as previously described (4D Analysis, Tomtec Gmbh, Germany) [14] and compared with the measurements obtained with both gated SPECT and RT3DE triplane imaging [see Additional file 2].

### Contrast protocol

The commercial available contrast agent Sonovue^® ^(Bracco Diagnostics, Inc.) is an aqueous suspension of stabilized SF_6 _microbubbles. The size of these microbubbles is between 1 and 10 μm, and their number is between 2 and 5 × 10^8 ^per mL. The solution was prepared according to the manufacturer's instructions and injected using a dedicated pump (Bracco, Italy) at an initial rate of 0.5 ml/min. The rate of bolus infusion was adapted for optimal visualization of endocardial border definition. The contrast-enhanced imaging was performed using predefined settings (low mechanical index < 0.5, gain 60%, compression 15%).

### Gated SPECT imaging

LVEF was calculated from the gated SPECT images using the full automatic program developed at Cedars-Sinai (GS-Quant, Siemens, Inc.).[15]

Myocardial perfusion SPECT gated in eight time bins were acquired 60 min after the injection of 925 MBq technetium-99m labelled sestamibi (MIBI) at rest using a dual-head gamma camera (ADAC Laboratories, Milpitas, California, U.S.A.) Detailed imaging and data processing have been reported previously [16]. The software algorithm segments the LV, estimates, and displays epicardial and endocardial surfaces and the valve plane for all images in the cardiac cycle and calculates the LVEDV and LVESV and derives the LVEF. Physicians interpreting the gated SPECT studies were blinded to the RT3DE results.

### Statistical analysis

Data are presented as mean ± SD. Two-tailed t-tests were used where appropriate. Linear regression with Pearson's correlation coefficient was used to assess correlations between RT3DE and MIBI gated SPECT data. Bland-Altman analysis was used to express agreement between RT3DE and gated-SPECT measurements. A subgroup of 20 randomly selected patients was studied for interobserver variability, which was determinated by using the same set of contrast RT3DE images measured by two separate sonographers. The interobserver variability in quantitative RT3DE LV volumes and LVEF was assessed by calculating the linear regression with Pearson's correlation coefficient and the limits of agreement between measurements made by use of Bland-Altman analysis between the two different observers [17]. A value of p < 0.05 was considered statistically significant. All statistical analyses were performed using SPSS (version 14.0) statistical software (SPSS Inc, Chicago, IL).

## Results

The baseline characteristics of the patients are summarized in Table 1. Most of the patients had a previous history of coronary artery disease.

**Table 1:** Clinical characteristics of the patient

	N = 38 (%)
Age (years)	70 ± 11
Male	20 (52%)
Hypertension	27 (71%)
Hypercholesterolemia	28 (73%)
Diabetes mellitus	6 (15%)
Tobacco use	15 (39%)
Prior myocardial infarction	8 (21%)
Prior percutaneous coronary intervention	25 (65%)
Prior coronary artery bypass surgery	19 (50%)
Acetylsalicylic acid	20 (52%)
Clopidogrel	11 (28%)
Statin	16 (42%)
Beta-blockers	12 (31%)
Angiotensin-Converting Enzyme Inhibitors	18 (47%)
Angiotensin receptors blockers	13 (34%)

### Left ventricular volumes and LVEF

The mean LVEDV of the group by scintigraphy was 143+65 mL (range: 66–323 mL) and was underestimated by triplane contrast RT3DE (128 ± 60 mL; p < 0.001) and less by full-volume contrast RT3DE (132+62 mL; p < 0.001). Limits of agreement with scintigraphy were similar for triplane andfull-volume modalities with the best results for full-volume (mean of the difference ± 2 SD: 5.2 ± 12.2). Results were similar for calculation of LVESV. By scintigraphy, the mean LVESV was 88 ± 62 mL (range: 26–264 mL) versus 75 ± 54 mL with triplane (p < 0.001) and 80 ± 57 mL with full-volume (p < 0.001). The mean LVEF was 44 ± 16% with scintigraphy and was not significantly different with both triplane contrast RT3DE (45 ± 15%) and full-volume contrast RT3DE (45 ± 15%).

### Accuracy and variability of contrast enhanced 3D echocardiography

**Figure 2:**
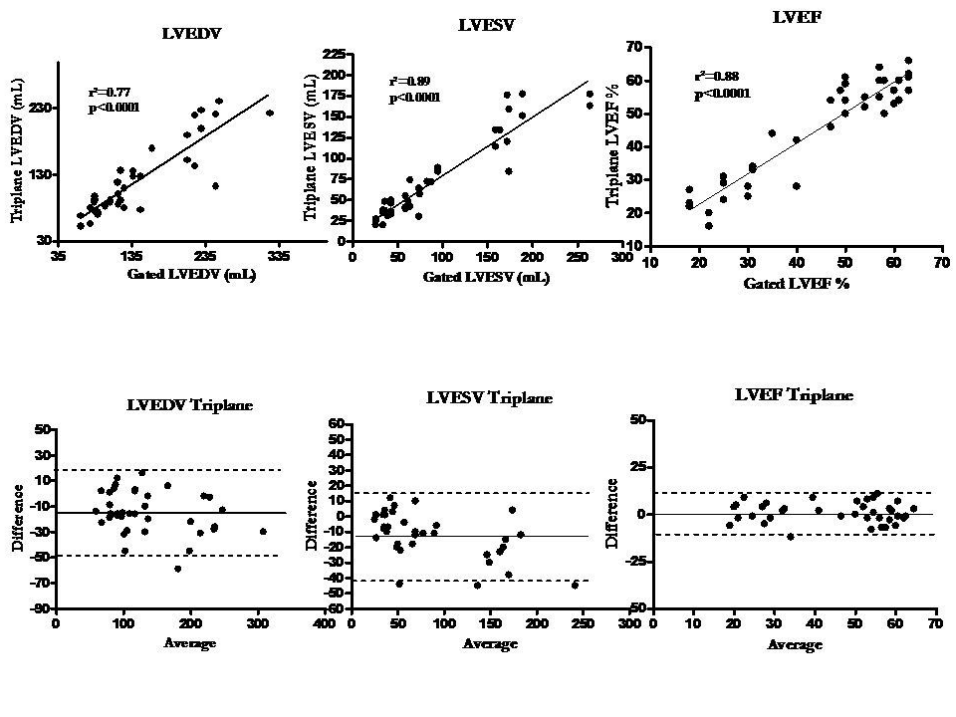
**Correlations and Bland Altman plots for EDV, ESV and LVEF with both RT3DE triplane and MIBI gated SPECT**.

**Figure 3:**
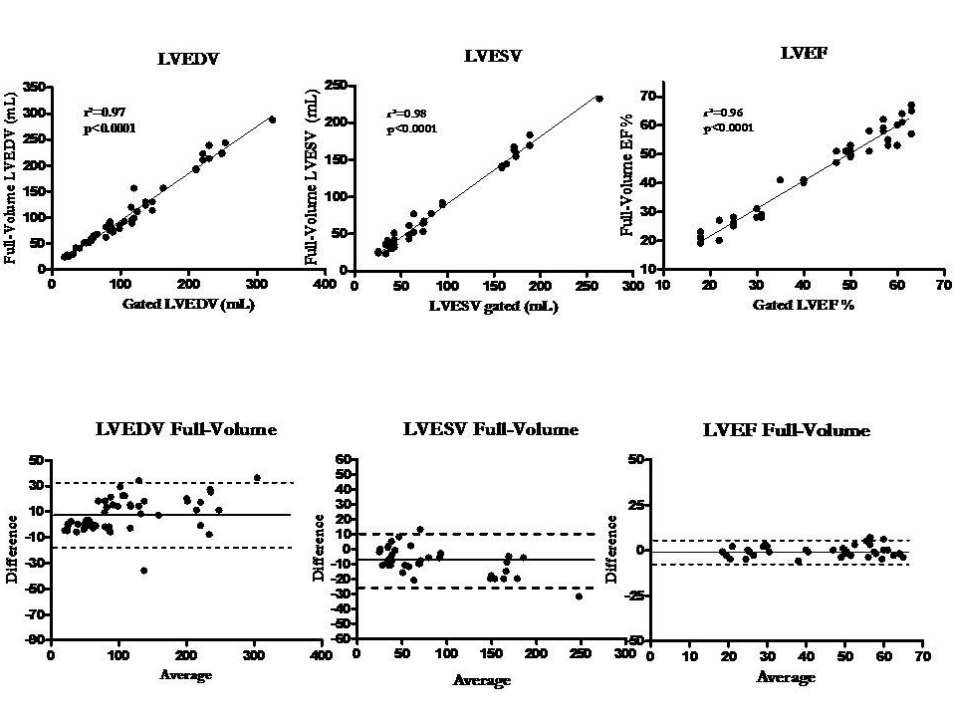
**Correlations and Bland Altman plots for EDV, ESV and LVEF with both RT3DE full-volume and MIBI gated SPECT**.

The correlation between contrast RT3DE triplane and full-volume with gated SPECT and the Bland-Altman plots are shown in Figure 2 and Figure 3 respectively. All measurements were strongly and significantly correlated for the different parameters when compared to scintigraphy. For LVEDV, LVESV and LVEF, the agreement was also good for both echocardiographic imaging modalities.

Triplane contrast RT3DE has shown a good correlation between two different observer's measurements for LVEDV (r^2 ^= 0.9; p < 0.0001), LVESV (r^2 ^= 0.9; p < 0.0001) and LVEF (r^2 ^= 0.9; p < 0.0001) respectively. Inter-observer agreement was also good for LVEDV (mean of the difference ± 2 SD: -0.09 ± 6.1), LVESV (mean of the difference ± 2 SD:-0.3 ± 7.8) and LVEF (mean of the difference ± 2 SD:-0.8 ± 7).

Regarding full-volume contrast RT3DE, there was also an excellent correlation between two different observer's measurements for LVEDV (r^2 ^= 0.9; p < 0.0001), LVESV (r^2 ^= 0.9; p < 0.0001) and LVEF (r^2 ^= 0.9; p < 0.0001) respectively. Inter observer agreement was also good LVEDV, LVESV and LVEF (mean of the difference ± 2 SD:-1.1 ± 2.8, -0.6 ± 2.5, -0.1 ± 2.3 respectively).

## Discussion

The results of the present study indicate that the combination of contrast with RT3DE triplane or full-volume allows the measurements of LV volumes and global systolic function in nearly all patients from an unselected population regarding baseline image quality. Our data further indicate an excellent accuracy of contrast RT3DE with a good correlation for LV volumes and LVEF assessment compared with MIBI gated SPECT, used as an independent reference. Although RT3DE triplane provides accurate evaluation of left ventricular function, RT3DE full-volume is superior to triplane modality in patients with suspected coronary artery disease. Finally, RT3DE with contrast shows a high interobserver agreement.

### Limitations of unhanced imaging

The main limitations of 2DE are tangential scanning yielding to LV foreshortening, low lateral resolution and geometric assumptions particularly in distorted ventricles. Other limitations are the low test-retest reproducibility and the underestimation of volumes compared to cineangiography, radionuclide ventriculography and MRI, especially for largest volumes. Using fundamental imaging, approximately 20% of resting echos demonstrate inadequate endocardial definition [18], defined as ≥ 2 segments not seen at baseline. While native tissue harmonic imaging enables better endocardial definition than standard fundamental imaging and reduces the number of patients with inadequate studies to 5–10%, contrast LV opacification still confers benefit over harmonic imaging [19].

### Limitations of unhanced 3D echocardiography

RT3DE compares favourably with reference methods [20,21]. However, when described in detail in the methods sections of these studies, the LVEF and cardiac volumes assessed by RT3DE were obtained among patients with "good" image quality and a percentage of patients (range 15 to 20%) were excluded because of an inadequate endocardial border delineation by the RT3DE [12,13,22-25]. A recent study comparing RT3DE without contrast with gated SPECT showed that poor echocardiographic image quality was associated with greater bias and larger confidence intervals [26]. In the present study, with the use of contrast, only 1% of the RT3DE were considered inadequate because we could not identify the endocardial border sufficiently well to render a meaningful manual tracing. This percentage of patients excluded for inadequate image quality is dramatically lower than previously published experience with RT3DE without contrast (range 4.3–13.8%) [9,13,27,28]. Moreover, the addition of contrast to 3D echocardiography has been shown to improve reproducibility and accuracy of this method when compared to unhanced 3D echocardiography relative to MRI [29].

### Gated SPECT compared to 3D echocardiography

Although MRI has been extensively validated and is known to give accurate estimates of left ventricular volumes and function, MRI is not readily available in most institutions; it is expensive, time consuming and it is not feasible in all patients [12,13,22-25,30]. Previous investigators have shown that MIBI gated SPECT imaging is a highly accurate method to determine these volumes [31-33]. Our results are strongly correlated with measurements derived from MIBI gated SPECT. Similar to report by other investigators, we found that ESV and EDV by contrast RT3DE mildly underestimate LV volumes as compared to the reference method [3,19,20]. These discrepancies may be accounted for by not including the volume under the mitral valve leaflets in the contrast RT3DE calculation of volumes and a possible overestimation of volumes by gated SPECT. The mitral valve opening and closure and consequently the timing of EDV and ESV can also be more difficult to determine when contrast is present. The underestimation of LV volumes with contrast RT3DE is less than expected, and the correlation with gated SPECT measurements is strong. This may be due to increase in image quality and to LV volumes increase secondary to use of contrast. A previous study has shown that the use of ultrasound contrast enhancement results in a sizable and significant increase in both end-diastolic and end-systolic RT3DE LV volumes, compared with non-contrast measurements [34]. The endocardium consists of sponge-like trabeculae with blood flowing in between them, the consequence of this anatomical fact appears to be largely underappreciated in quantitative RT3DE analysis. The intertrabecular space may actually comprise a large part of the true LV cavity volume – a volume that traditionally remains undetected, as the LV trabeculae would be indistinguishable from the LV wall if contrast were not used. However, other authors have reported underestimation of LV volumes with contrast [35]. Although we didn't compare our LV volumes measurements with or without contrast, our data indicate that LV volumes measurements with contrast RT3DE still underestimated volumes compared to the MIBI gated SPECT.

### Limitations of 3D echocardiographic modalities

The main limitations of full-volume RT3DE are the need for expertise, the off-line and time consuming analysis and the possible undersampling secondary to low frame rate (potential underestimation of LVEDV and overestimation of LVESV). Given that the images were obtained at rest, the temporal resolution of contrast RT3DE was similar to that obtained with MRI and superior compared to gated SPECT.

On another hand, full-volume dataset may be insufficient to capture the entire LV volume of a large heart but this might be alleviated by ongoing technical improvements."

Compared to a previous study [26] for a group of patients who seem to have similar distribution of ventricular volumes, the average biases for the end diastolic volumes appeared to be much greater with the triplane modality. The biases would be expected to be lesser in this group compared to this earlier study because of the use of contrast echocardiography. This was probably due to the relatively small number of imaging planes used to record the volumes. The bias was less important compared to gated SPECT when using RT3DE full-volume measurements. Our study also showed that contrast RT3DE triplane was a reproducible technique with an excellent correlation between two different observer's measurements which confirms previous studies about RT3DE showing that this technique is less dependent on operator skills, can help to compensate for sonographer inexperience and has a higher reproducibility than 2DE [16-18]. Contrast RT3DE full-volume has been shown to be superior to RT3DE triplane for reducing the scatter and by improving accuracy and reproducibility [36]. Although it was confirmed in the present study, it remains more time consuming, requires additional software and gating. The latter may limit application of this technique in patients with arrhythmias and requires stitching of data collected during four consecutive beats. Although less accurate than full-volume, contrast RT3DE triplane has sufficient accuracy to provide a close approximation for a valuable measurement of volumes and LV function compared to gated SPECT. A new generation of echo machines has been recently released, allowing the acquisition of the full-volume data set in one beat with automated delineation of the endocardial border. Pending validation, this should be the standard for measuring LV volumes and LVEF in the upcoming years.

### Clinical utility

The evaluation of LV volumes and LVEF is sought for major decisions in cardiology. The combination of contrast with RT3DE offers the unique opportunity to alleviate the main limitations of 2DE and to be used in unselected population. RT3DE does not have significant limitations in clinical application such as exposure to ionizing radiation (the dose of a technetium sestamibi scan corresponds to 500 chest x rays and to an extra risk of cancer of about 1 in 2000 exposed patients for one single examination), exposure to radiographic contrast material, cost, invasive nature, or contraindications such as the presence of implanted metallic objects or claustrophobia. Moreover, contrast RT3DE has the advantage to be a portable and versatile technique with a high spatial and temporal resolution that can be performed at bedside. Therefore it is the ideal tool making serial study of LV volumes and LVEF practical.

### Limitations of the study

Contrast was used in all patients without any selection. Even patients with good quality image had received contrast. We did not analyze the impact of contrast in function of baseline image quality. Therefore, the requirement for contrast for all RT3DE studies can not be extrapolate from the present study. However, it has been shown that increase of inter-observer reproducibility with contrast enhanced left ventricular opacification was not restricted to patients with poor baselineimage quality (11), so the benefit of contrast may not completely relate to improved endocardial resolution. The avoidance of off-axis image projections with contrast enhanced left ventricular opacification in patients with good resting images likely explains the better accuracy of LV and LVEF calculations with RT3DE.

The study patients were all in sinus rhythm; thus, the results cannot be generalized to patients with arrhythmia. The reason for excluding these patients was mainly the risk of inadequate gated SPECT quality.

## Conclusion

Contrast 3D echocardiography allows an accurate assessment of LVEF compared to the LVEF measured by SPECT, and is reproducible. Although RT3DE triplane provides accurate assessment of left ventricular function, RT3DE full-volume is superior to triplane modality. However, both methods have sufficient accuracy to provide a close approximation for a valuable measurement of volumes and LV function compared to gated SPECT in patients with suspected coronary artery disease. This technique provides a quick, inexpensive, portable and accurate alternative for LV size and function analysis and can be considered as the method of choice for the daily measurement of LVEF, especially when serial measurements are required.

## Competing interests

The authors declare that they have no competing interests.

## Authors' contributions

BC, DH and SD participated in the design of the study and performed the statistical analysis. BC and PL conceived of the study, and participated in its design and coordination. SW, PM, BC and EL participated to the acquisition and the analysis of the images. MM and SH participated to the elaboration of the manuscript. All authors read and approved the final manuscript.

## Supplementary Material

Additional file 1 **Contrast enhanced triplane real time 3D echocardiography**. Example of contrast triplane real time 3D echocardiography. Click here for file

Additional file 2 **Contrast enhanced full volume real time 3D echocardiography**. Example of 9 slices contrast full volume real time 3D echocardiography. Click here for file
